# The Roles of Family and School Members in Influencing Children’s Eating Behaviours in China: A Narrative Review

**DOI:** 10.3390/children9030315

**Published:** 2022-02-25

**Authors:** Jianlin Xu

**Affiliations:** School of Geography, University of Leeds, Leeds LS2 9JT, UK; fsjxu@leeds.ac.uk

**Keywords:** eating behaviour, family and school members, children, social learning, social interaction, family relationships, China

## Abstract

This review explores the influences of family and school members on children in China, in order to promote healthy eating behaviours among children and prevent childhood malnutrition in the Global South. Family members and school members are defined as parents, guardians (such as grandparents and other relatives), siblings, peers, and teachers. A search of four databases returned 94 articles, 18 of which met the eligibility criteria. Most of the included studies were from mainland China; a few were from Hong Kong and Taiwan. More quantitative than qualitative studies were found, among which, cross-sectional studies were dominant. The 18 papers included in the study explored the influences of family members and school members on the eating behaviours of children, based on seven themes: (1) social–demographic characteristics, (2) food intake of parents, (3) nutritional knowledge and health awareness of family or school members, (4) parents’ perceptions of their children’s body weight, (5) feeding strategies of family members, (6) family relationships, and (7) intergenerational differences of caregivers. In the current analysis, parental education levels, mother’s occupation, health awareness of parents and teachers, and positive feeding styles, such as encouraging healthy eating and controlling overeating, were positively correlated with the healthy eating behaviours of children. Meanwhile, healthy eating behaviours of children were negatively associated with caregivers’ lack of nutritional knowledge, misperception of weight, instrumental and/or emotional feeding, and working on nonstandard shifts. More related research using cross-disciplinary approaches is needed and there should be more discussions about how teachers, siblings, and peers affect the dietary behaviours of children.

## 1. Introduction

An individual’s long-term eating habits is established in childhood—a vital period that may influence one’s future risks of metabolic diseases, being overweight or obese, and other nutrition-related illnesses [1,2,3]. However, the majority of children’s (“children”, hereafter, refers to kids under 18 years old) diets worldwide are unsatisfactory or unhealthy [4,5,6,7]. This is evidenced by the trend that the number of overweight and obese children worldwide has grown ten-fold in forty years [8]. Globally speaking, there are around 42 million overweight children, more than 35 million are from developing countries [9]. In China, according to the China Nutrition and Chronic Diseases Status Report (2020), 19% of children aged 6–17 and 10.4% of children under 6 years old were overweight and obese, respectively. This shows that "overweight and obesity" among Chinese children is gradually becoming more severe and the incidence of children with chronic diseases is on the rise.

However, significant inequalities exist between poor rural areas and more affluent urban areas in China. While the dietary composition of Chinese children has changed rapidly and childhood obesity is rising, undernutrition is still a threat that might hinder the nutritional improvement for generations in some geographic regions in China [10]. Children’s undernutrition (usually referred to stunted growth and being underweight) remains a prominent problem in China’s underdeveloped regions and rural areas [10,11,12]. Due to rapid economic reform and urbanization, China, as with other countries in the Global South, has experienced a transition from undernutrition to the coexistence of overnutrition and undernutrition—a so-called “double burden” phenomenon [11,13,14].

Many nutrition-related diseases might be prevented or improved through changing lifestyles, particularly by adopting well-balanced diets [15]. In China, family and school are the most important environments for children to live and socialize in; therefore, individuals in these places strongly impact the behavioural patterns of children, including their eating behaviours, which have life-long developmental effects (e.g., pertaining to their health). From a sociological perspective, of all the "stimuli" in a child’s environment, other people—particularly those who are closest to the child—will arguably have the greatest effect on the child’s behavioural patterns [16]. Albert Bandura, a psychologist who proposed the social learning theory and examined the influence of role models and imitation regarding childhood aggression [17], revealed that, for many people, behaviour is shaped through observation and imitation of other people. In the family environment, it is commonly believed that a child’s dietary behaviours is significantly influenced by his/her parents, who often act as gatekeepers and role models that the child will follow and learn behavioural patterns from [18,19]. Moreover, individuals in the same household often eat together; this is especially true for children, who can also be affected by other family members, such as grandparents, siblings, and other relatives in their early lives [20]. Meanwhile, a number of studies [21,22,23] reported that school also contributes to a child’s food choices. In a school setting, teachers who care more about their own dietary health also tend to be more interested in the health of their students. Moreover, it is believed that teachers could also act as models to improve the healthy behavioural patterns of students [24,25]. In addition, because of the pressure from peers, children may have to buy and consume unhealthy foods in schools [21] to fit in.

Given the increasing concerns about the nutritional statuses of children [26,27], many studies have shown strong international interest in exploring the eating behaviours of children, including their associations with different social members. Although studies in this area have rapidly increased, there are two noteworthy research gaps. Firstly, most studies have focused on family environments and targeted the correlation between the dietary behaviours of parents and their children [20]. However, the family environment is just one of many complicated and intersecting factors that impact the eating behaviours of children [28,29,30]. For instance, it is common for most school-age children, particularly those from the growing number of families with two working parents, to consume one or two meals at their schools (during term time). Moreover, as children grow older, they become more susceptible to their peers when making food decisions [20]. Consequently, it is pertinent to understand the influences of all main family members (parents, guardians, such as grandparents and other relatives, siblings) and school members (peers and teachers) on a child’s eating behaviour. Another gap is that the majority of studies focus on western countries, particularly North America and Europe. There are a limited number of studies examining how family and school members affect the dietary behaviours of children within the context of the unique Chinese food environment and culture.

Therefore, this narrative review aims to probe the roles of family and school members in impacting children’s eating behaviours in China. There are three primary purposes of this review: (1) to summarise the results and implications of the included studies conducted in China; (2) to identify important factors within the families and schools that may affect the eating behaviours of children; (3) to provide useful insights for relevant stakeholders, such as policymakers, government agencies, agri-food industries, and public health institutions, to promote healthy eating behavioural patterns among children in the Global South.

## 2. Methods

### 2.1. Search Strategy

The literature search was conducted on four electronic databases—Scopus, Web of Science, EBSCO, and PubMed—in October 2020 and updated in February 2021. The following combinations of keywords were used: parent*, mother, father, sibling, caregiver, carer*, grandfather, grandmother, family member, peer*, friend, teacher, eat*, diet*, child*, adolescent*, China, Chinese, Hong Kong, Taiwan, Macau. Originally, a total of 94 records were identified on the subject: 41 in EBSCO, 45 in Web of Science, and 8 in PubMed. No search date restriction was applied, as studies on this topic in the context of China have just emerged in recent years, with the earliest literature based on search results appearing in 2008.

### 2.2. Study Selection

**Figure 1:**
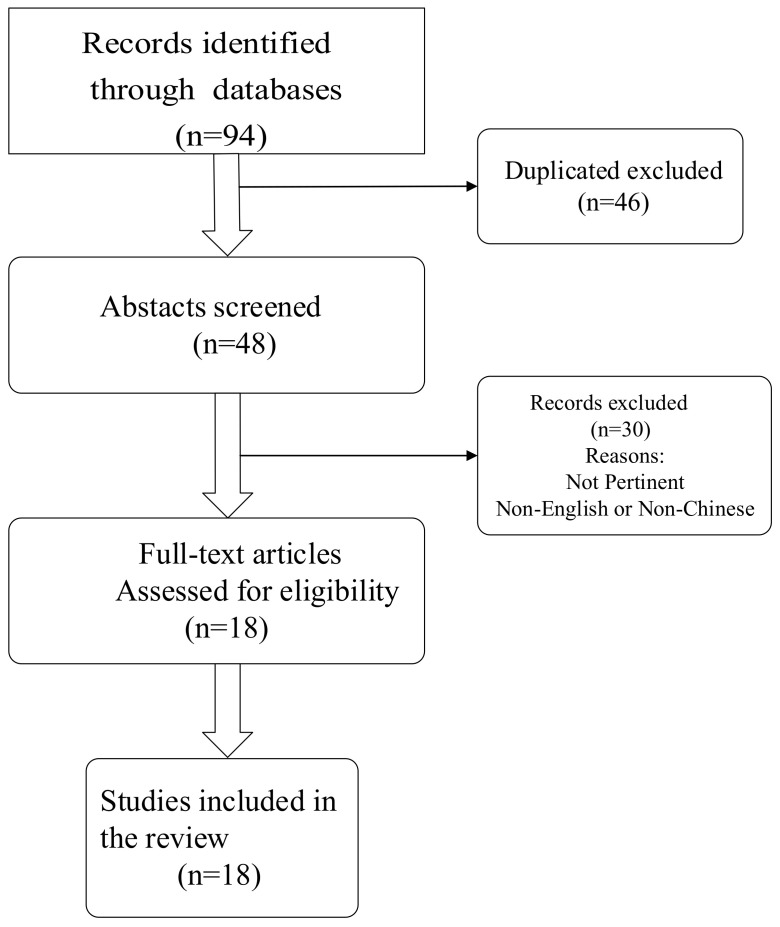
The flow chart of article selection process for this study.

After using the search strategy and removing duplicates, 48 papers were obtained. Then, these papers were further selected based on the eligibility criteria: language in English or Chinese; published in peer-reviewed journals; available in full-text; reported whether family and/or school members impacted the eating behaviours of children, as well as what factors could explain the effects. In total, 18 of 94 studies identified in the review met the eligibility criteria. The flow chart of the article selection process for this review is shown in Figure 1.

## 3. Results

### 3.1. Descriptive Information

The summary of the descriptive statistical information of the articles included is shown in Table 1. Descriptive results of the articles were calculated manually during the analyses. The included studies were classified according to study design and research objects.

Table 1 shows that, of the 18 studies, over half of the studies (N = 10, 56%) focused on parental influence over the eating behaviours of children, with only 1 study examining both parental and teacher influence. No studies so far have looked at the influence from siblings and peers. Location wise, 14 studies were conducted in mainland China, 3 in Taiwan, and 1 in Hong Kong. The majority of studies used a quantitative approach (N = 15, 83.2%), with 13 adopting a cross-sectional design. Only 3 studies used a qualitative approach by applying methods of multiple cases studies, food diaries, and semi-structured interviews for data collection.

In regard to the study population’s age range, 6 (one-third) of studies focused on pre-school aged children (2–7 years), with another 6 on children of mixed ages—both pre-school and school-aged children; 5 studies focused on school-age (6–18 years) children only, and 1 study looked at the eating behaviours of adolescents.

The included studies measured children’s eating behaviours, according to their different methodologies. Studies using a quantitative approach (15 of 18 studies, 83.2%, among which, the majority were cross-sectional (13 of 15 studies)) usually measured children’s eating behaviours by questionnaires on food frequency as well as food knowledge and attitude, reported either by caregivers or children themselves. For research that used qualitative research methods (only 3 articles), they collected data using multiple cases studies, food diaries, and semi-structured interviews, respectively.

### 3.2. Main Findings

Characteristics of the included studies are shown in Table 2. The method of “thematic analysis” [31], which could identify, analyze, and report patterns (themes) within data were applied to this study for summarizing the results of the included papers. The main findings of the included studies are summarized in the following seven themes:

Theme 1: Social–Demographic Characteristics

*Sub-theme A: social–demographic characteristics of family or school members.* In most studies, social–demographic variables, such as education, gender, occupation, as well as monthly income of the family and school members, were considered for their impacts on children’s eating behaviours. For example, the study by Hu et al. [32] argued that preschoolers whose fathers had less educational degrees (basic education vs. higher education) were more likely to show problematic eating behaviours, such as picky eating. Besides, it demonstrated that parental occupation and monthly household income were significantly correlated with children’s dietary behaviour levels. Moreover, He et al. [33] conducted a study involving a sample of 11,270 parents and 1378 teachers, noting a correlation between more than one parent having a high school education and a child’s calorie-rich eating habits; these associations were negatively correlated among urban school-aged children and were positive in rural areas. This may be because of the effects of other confounders, such as economic level and nutritional knowledge between education level and a child’s high-calorie diet. Generally, parents with education levels higher than high school, in cities, were more likely to have college degrees or above. Therefore, they had higher economic incomes and nutritional knowledge, which resulted in their controlling a child’s high-calorie diet. However, in rural areas, parents with more than a high school education may have had a relatively high income, but still lacked nutritional knowledge, so they might have given their children more pocket money to buy high-calorie foods. The same authors [33] explored the influence of both parents and teachers on school-age children. The results showed a positive association between at least one parent with more than a senior high school education and healthy dietary behaviours in the kids, while there were no correlations between the education levels of teachers and eating behaviours of children. This may be due to a large gap between the parents’ educational levels, while teachers’ education degrees are usually similar. Furthermore, some researchers studies [34,35] explored the influences of parents’ work characteristics, residences, and religiosity. A study [35] involving a Taiwan sample suggested that children with at least one parent who worked irregular hours were more likely to skip breakfast and have unhealthy, non-core food intakes every day, compared to kids whose parents had standard working shifts (which means they had regular commuting hours). Another cohort study [36] tracking from 1991 to 2006 reported that rural children had more traditional diets than those who lived in cities. In addition, the research [34] conducted a bivariate correlation analysis between the frequency of parents’ participation in religious activities and food intake in children, in Ningxia province, China. It revealed that, among those of Hui ethnicity, the frequency of a mother’s religious attendance was statistically negatively correlated with a child’s vegetable consumption.

*Sub-theme B: social–demographic characteristics of children.* Some studies demonstrated that the age and gender of children were important influencing factors for their eating behaviours. One study [32] collected 1781 questionnaires among parents of preschoolers; the kindergartens of Chongqing claimed that the child’s age was negatively correlated with dietary behaviour levels. The reason may be that the study only targeted preschool children. Preschoolers’ diets are chosen by the parents, when the kids are young; at that time, parents can control what their kids eat; however, as these children get older, they will have more autonomy to choose their preferred foods and they are likely to purchase some unhealthy food. Interestingly, another study [37] pointed out that, compared to female kids, it was more likely for carers to underestimate the body weight of male children; thus, they had less control over the diet intakes of boys.

Theme 2: Parental Food Intake

In the family environment in China, parents serve as role models for their children’s early eating behaviours and even take responsibility for their food choice decisions. Therefore, most children will adopt and imitate their parents’ dietary patterns. In the study [38], researchers collected data on participants’ food intakes based on the China Health and Nutrition Survey (CHNS) 2011. These researchers divided “food intake” into 10 categories: grain, vegetable, fruits, meat, beer, fish, egg, dairy, drink, and snack. They found a statistically significant positive correlation (*p* < 0.01), for each kind of food, between the food intake of children and their parents. Moreover, the influence of a mother’s food intake on her children was stronger than the father’s (except for the category of drink). Furthermore, this study reported that the correlations were more significant in the rural regions of China than urban regions. Additionally, it was found that overweight and obesity in children were relevant to parental food intake; the association was stronger in young children below 12, but became weaker among children between 13 and 18. Another study [39], using longitudinal data from the CHNS (1991–2009), found positive parent–kid associations for diets. Meanwhile, the amount of energy Chinese children got from animal-based foods, non-homemade meals, and snacks had increased by 10% in a 20-year period. The findings of this study show a need for interventions, aimed at both children and parents, regarding healthy eating behaviours in the family context.

Theme 3: Family or School Members Nutritional Knowledge and Health Awareness

Zeng et al., to investigate the associations between caregivers (including parents or guardians, such as grandparents, uncles, aunts, etc.) and their children’s dietary behaviours, conducted a study [40] involving 3361 children aged 2–7 years old in Chinese rural areas. They developed a questionnaire using 10 nutritional questions to measure caregivers’ nutritional knowledge levels, finding that, having a caregiver whose nutritional knowledge was measured at a lower level (correctly answered less than 6 out of the 10 questions) was associated with more frequent unhealthy dietary behaviours among the children. These behaviours included resistance to milk, fussy eating, skipping breakfast, or eating irregular meals. Another study [33] revealed that health awareness and active health attitudes of teachers were positively correlated with their students’ healthy dietary behaviours, while unhealthy eating behaviours were contrary.

Theme 4: Parents Perceptions of Their Children’s Body Weight

Parents should evaluate the body weights of their kids, objectively, if they want to promote positive improvements in their diets and adopt appropriate diet control strategies, accordingly. A series of studies [32,37,41] examined the relationship between parents’ perceptions of their sons’ and daughters’ body weights and their eating behaviours. Parental inaccurate cognition of a child’s weight was more likely to result in the child’s unhealthy eating behaviours compared with a child whose parents had a correct perception [32]. A study by Tang et al. [37], involving 364 Chinese preschoolers in Changsha, a city in central China, showed that it is more probable for caregivers to underestimation their children’s body weight in urban areas than rural areas. If caregivers have boys, or if they come from low- or middle-income families, it is more possible for them to have inaccurate perceptions of the overweight–obese status of their children. Furthermore, this study showed that caregivers who underestimated the weight of their children were less likely to be concerned about the nutritional statuses of their children, control the food intake of their children, and may have had children with poor appetites. In addition, another study [41] that examined maternal influence provided evidence that a mothers’ body weight concerns, and the psychological characteristics associated with these concerns, positively related to the dietary attitudes of their kids.

Theme 5: Family Members’ Feeding Strategies

The feeding strategies of caregivers also crucially influence the food preferences of children. A study conducted by Yuan et al. [42] among preschool-aged children in Jinan and Xi’an, China, reported a link between the feeding types of caregivers and their kids’ eating behaviours. They found that caregivers who encouraged healthy eating, responsible feeding, supervision of food intake, and controlled feeding were more likely to promote healthy dietary behaviours among their children, particularly more initiative eating and less frequent unhealthy eating behaviours, such as emotional eating and food fussiness. Besides influencing the eating habits of children, another study [43] suggested that caregivers’ feeding styles could also affect the dietary intakes of children. Caregivers’ instrumental and/or emotional feeding patterns may relate to insufficient intake of fruits, vegetables, and breakfast in children, showing a positive correlation with high-calorie food consumption. In contrast, children were more likely to eat fruits, vegetables, dairy, and breakfast regularly if their caregivers adopted encouraging feeding approaches. In addition, this study showed that children tended to eat more fruits, vegetables, breakfast, and fewer energy-dense foods if their caregivers controlled their diets. The enlightenment from the results of these studies is that the feeding strategies of caregivers are important influencing factors that should be considered in related studies concerning children’s eating behaviours. Although existing studies on this topic, in a Chinese context, suggest that efforts to prevent malnutrition in a child may benefit from targeting not just what a child eats, but how he/she eats [44], there is a lack of comparative research on the differences between food parenting strategies of different caregivers, such as fathers and mothers, which may contribute to improving the interventions, by targeting the role each parent plays in promoting a child’s healthy eating habits [45] in China.

Theme 6: Family Relationships

Tensions in relationships between family members, such as family conflict, can also affect a child’ eating behaviours. In a qualitative study [46] using multiple-case studies, the researcher identified three different kinds of parent–child conflicts from the psychological consultation data collected among 10 families whose daughters suffered from eating disorders: power struggle and relationship control between generations, becoming mature or remaining childish, and pursuing personal dreams or meeting parents’ expectations. This study further analyzed that parents tended to follow a therapist’s guidance, believing that, for their kids, food was the most effective medicine. Therefore, these parents could insist on refeeding their kids. However, this kind of persistent behaviour could easily be regarded by children as a means of control or coercion. Instead, this may provoke these children to become more stubborn in their eating resistance. Similarly, Zhu et al. [47] conducted a study involving 594 high school students, reporting that the control and negative emotions of parents significantly correlated with children’s emotional eating patterns. It was shown that parental control was the mediator between negative emotions and emotional eating, which accounted for a 52.6% explanation degree of the relationships between them. In addition, the study conducted by Chao et al. [48] among 600 Taiwanese children suggested that children’s picky eating patterns were often related to parents’ inappropriate interactions, such as threatening, over-snacking and inappropriate nutrient supplication. The study argued that parents who were anxious about their children’s diets and psychological development were more likely to identify inappropriate eating behaviours in children who were picky eaters.

Theme 7: Caregivers’ Intergenerational Differences

It is a common arrangement for children to be cared for by their grandparents in the childcare culture of China [49]. Therefore, Chinese grandparents play an important role in multigenerational families [50]. Some studies [51,52,53] compared the effects of intergenerational differences of caregivers and the eating behaviours of children. According to Wang et al. [51], when it comes to feeding, it is more common for grandparents to adopt a permissive or indulgent way, while parents show more gentle persuasion or use treats and bribes. Besides, the study [52] conducted by Su et al. among 1–4 year old "left-behind" children (those who were under the care of grandparents because their parents were both working outside of the village) in Anhui province reported that left-behind children might become the higher risk group in regard to childhood malnutrition, compared to those children with parents at home. One possible reason may be the unhealthy feeding patterns offered by the grandparents of these left-behind children. Another qualitative study [53] in a rural township of Henan province presented similar results, they found that left-behind children who were intergenerationally fed—particularly those cared for by grandparents who lived through China’s Great Famine in the 1960s—were more likely to suffer from malnutrition.

**Table 2:** Summary of the eligible studies.

ID	Year	Study Design	Sample Size	Identified Theme(s)	Reference
1	2019	Cross-sectional	956 preschool children aged 3-6 and their caregivers	Family members’ feeding strategies	Yuan et al. [42]
2	2015	Cross-sectional	4553 preschoolers	Family members’ feeding strategies	Lo et al. [43]
3	2019	Cross-sectional	1781 parents of preschool children	Social–demographic characteristics; parental perceptions of the body weights of their children	Hu et al. [32]
4	2012	Cross-sectional	3361 rural caregivers and their children, aged 2–7	Family or school members’ nutritional knowledge and health awareness	Zeng et al. [40]
5	2014	Cross-sectional	11,270 fourth to sixth grade students, 11,270 parents, and 1348 teachers	Social–demographic characteristics; family or school members’ nutritional knowledge and health awareness	He et al. [33]
6	2020	Cross-sectional	1631 children and their parents	Parents’ food intake	Tang et al. [38]
7	2017	Cross-sectional	600 caregivers whose children aged 1–10	Family relationships	Chao & Chang [48]
8	2011	Longitudinal	966 mother-child pairs (children aged 3–5 at baseline)	Social–demographic characteristics	Dearth-Wesley et al. [36]
9	2008	Cross-sectional	241 children aged 10–13	Parents’ perceptions of their children’s body weight	Tao & Zhong, 2008 [41]
10	2018	Cross-sectional	364 urban children aged 2–6	Social–demographic characteristics; parental perceptions concerning the body weights of their children	Tang et al. [37]
11	2018	Cross-sectional	18,046 children	Social–demographic characteristics	Wu [35]
12	2020	Cross-sectional	1690 students aged 6–18	Social–demographic characteristics	Wu et al. [34]
13	2014	Cross-sectional	594 high school students aged 15–18	Family relationships	Zhu et al. [47]
14	2016	Longitudinal	5201 parent–child pairs (children’s age: 7–17)	Parents’ food intake	Dong et al. [39]
15	2012	Cross-sectional	424 left-behind children aged 1–4	Caregivers’ intergenerational differences	Su et al. [52]
16	2008	Qualitative	10 children (and family) undergoing therapies for eating disorders	Family relationships	Ma [54]
17	2015	Qualitative	26 children (21 of which were left-behind children) aged 6–12, and their caregivers	Caregivers’ intergenerational differences	Zhang et al. [53]
18	2020	Qualitative	Primary caregivers of elementary school children from 23 households in 4 remote areas of Taiwan	Caregivers’ intergenerational differences	Wang et al. [51]

## 4. Discussion

### 4.1. Positive and Negative Aspects of Family and School Members’ Effects

This review identified five factors that have positive effects on the dietary behaviours of children—caregivers’ high education level [32,33], mother’s occupation [32], good parents and teachers’ health awareness [33], positive feeding style, such as encouragement of healthy eating [42,43], and controlling overeating [43]. However, there are some inconsistencies in these research results showing positive impacts. In one study [33], there was a positive correlation between kids’ healthy dietary behaviours and more than one parent having an education degree beyond senior high school, while another study [41] showed no correlation between the mother’s education level and her children; Hu et al. [32] claimed that only the father’s educational status had an influence.

The negative impact of family and school members was observed in some studies. They showed that caregivers’ lack of nutritional knowledge and improper weight perceptions might result in unhealthy eating behaviours of the children. The study by Zeng et al. demonstrated that, in rural China, a low level of nutritional knowledge among caregivers was significantly associated with unhealthy eating behaviours in children, including disliking milk, picky eating, skipping breakfast, etc. [40]. Besides, if a caregiver underestimate a child’s body weight, the carer might be less likely to restrict the child’s diet, and not worry about whether the child is obese, and it is more probable for the child to have a poor appetite [37]. Some studies have also reported a connection between inappropriate parental interactions and the eating problems of children [48]. Caregivers’ instrumental and/or emotional feeding styles may relate to a child’s insufficient intake of fruit, vegetables, and breakfast, showing a positive correlation with high-calorie food consumption [43]. Moreover, children tend to miss breakfast every day and overconsume unhealthy snacks if their parents work non-standard shifts [35]. Meanwhile, there also exist some ambiguities in the studies presenting negative influences. For instance, the authors of a study [40] claimed that low nutritional knowledge in caregivers results in unhealthy dietary behaviours of children, such as disliking milk, picky-eating, skipping breakfast, or having irregular meals, but there was no correlation with snacking. In another study [38], researchers investigated the correlations between parental food intake and overweight and obesity in their kids, but did not provide a reasonable explanation for why the connections were stronger in rural China compared to Chinese urban areas.

**Figure 2:**
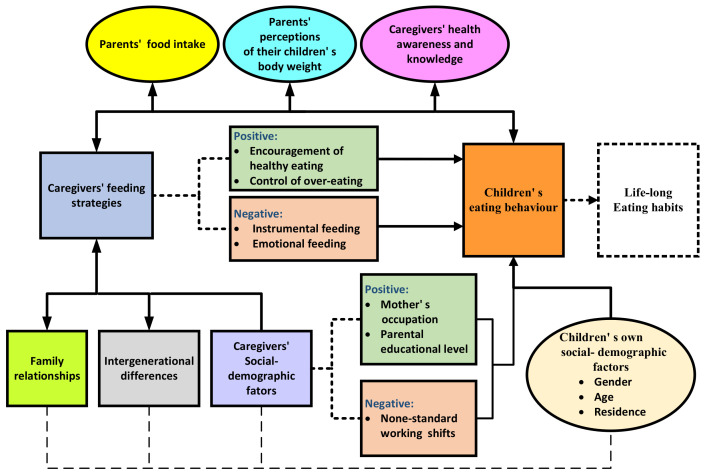
Relationships between the seven themes and eating behaviours of children.

The relationships between the seven themes and eating behaviours of children from the 18 included literature are shown in Figure 2.

### 4.2. Group Identity

Human eating behaviours are not only biological behaviours, but also social practices. In society, food has many symbolic meanings, and it is also a means for people to establish and express relationships between one another [54]. Enlightened by social learning theory, we can extend the understanding of our results. The social learning theory was proposed by psychologist Albert Bandura in 1977. It emphasised the interactions between humans and the environment, these interactions include two main types, one is direct social interaction (e.g., direct communication) and the other is indirect social interaction (e.g., observation) [55]. When people make decisions, they may be impacted either directly by their acquaintances or indirectly by their social networks [56].

Family and school members play key roles in the everyday lives of children, influencing their eating behaviours. Besides parents—siblings and peers are some of the most crucial active social agents that affect the behavioural patterns and attitudes of children [57,58]. However, based on this review, most studies only discussed parental impacts on a child’s eating behaviours, while very few studies examined the impacts of both parents and teachers. Studies that explore and explain the influences of siblings and peers are lacking in the context of China. In fact, peers provide an important social context in which children could gain their sense of identity through compared with others [59]. Children who consume particular foods or adopt similar eating habits to their siblings and peers might be a way for them to express “belongingness” [60,61]. In particular, as some studies have shown, people in late childhood and adolescence have strong desires to be accepted by their peers [62]. Therefore, there is a need for future empirical research to investigate the associations of social interactions with siblings and peers, the aspects of group identity, and eating behaviours of children.

### 4.3. The Importance of Indirect Social Interaction

Most of the studies included in this review analyzed the correlations between family members and eating behaviours of children from the direct social interaction perspective. For example, some studies have simplified the influence of parents on the eating behaviours of their children to the impacts of parental food intake and feeding styles. There were limited discussions from the perspective of indirect social interaction. Only a few studies reported on the indirect impacts, such as parental work characteristics [35], religiosity [34], and family relationships [46]. Indirect social interaction, according to Bandura, is defined as children learning how to behave by observing others in social environments [63], or by connecting with others in their own social networks [56]. In a Chinese social context—the influence of family and school members on the eating behaviours of children is not only done by explicit information verbally exchanged between individuals (direct social interaction), but also by observing other people in different micro-social systems [63]. Unfortunately, indirect influencing has been less studied. Hence, it is necessary to add more studies from this perspective.

### 4.4. Methodological Considerations

The majority (83.3%) of the included studies were primarily based on quantitative methods, especially cross-sectional design (72.1%). Some of the quantitative studies analyzed the impacts of family members on the dietary behaviours of children by simply comparing food intake or social–demographic characteristics between them. Strong causal relationships of direct, and in particular, indirect influencing factors on the dietary behaviours of children, are difficult to draw from these types of studies. In addition, most studies in this direction are empirically research-based in certain regions of China; the studies that systematically examined the validity of individual studies and used meta-analyses to test whether those studies produced consistent results are limited. Therefore, more cross-disciplinary approaches, qualitative methodologies, as well as systematic and meta-analysis review studies are needed in this area.

## 5. Conclusions

To conclude, family and school members play active and important roles in shaping the eating behaviours of children in China, although it is difficult to disintegrate these complex factors, including how family and school members influence the eating behaviours of children, because they are reciprocally interacting. This study identifies seven themes that may explain Chinese family and school member influences on the eating behaviours of children. Moreover, quantitative research, particularly a cross-sectional study, is the dominant research method used in these studies (72.1%). Qualitative research is limited, but provided some insights into the associations between family conflicts, caregivers’ intergenerational gaps, and adolescent eating disorders in China. Future research should incorporate more cross-disciplinary research to add greater insight to the body of evidence. In addition, to better understand the underlying sociocultural mechanisms that affect the eating behaviours of children, we need to consider more potential, indirect social interactive factors embedded in the social contexts of the Global South, which may vary from developed regions.

## Figures and Tables

**Table 1:** Descriptions of the included studies.

Variable	Total-n (%)
Total-N	18
**Regions studied**	
China mainland	14 (78%)
Taiwan	3 (17%)
Hong Kong	1 (5%)
**Study Design**	
**Quantitative**	
Cross-sectional	13 (72.1%)
Longitudinal	2 (11.1%)
**Qualitative**	
Multiple cases studied	1 (5.6%)
Food diaries and semi-structured interview	1 (5.6%)
Semi-structured interview	1 (5.6%)
**Study Population**	
Pre-school age children	6 (33%)
School-age children	5 (28%)
Children of mixed ages	6 (33%)
Adolescents	1 (6%)
**The type of influence on eating behaviours**	
Only parents (10 in total, 2 of whom studied mothers only)	10 (56%)
Caregivers	7 (39%)
Both parents and teachers	1 (6%)
